# Yarning about oral health: perceptions of urban Australian Aboriginal and Torres Strait Islander women

**DOI:** 10.1186/s12903-020-1024-x

**Published:** 2020-02-03

**Authors:** Kaley Butten, Newell W. Johnson, Kerry K. Hall, Maree Toombs, Neil King, Kerry-Ann F. O’Grady

**Affiliations:** 1 Queensland University of Technology, Institute of Health & Biomedical Innovation, Centre for Children’s Health Research, 62 Graham Street, South Brisbane, Qld, Brisbane, 4101 Australia; 2 Menzies Health Institute Queensland and School of Dentistry and Oral Health, Gold Coast Campus, Griffith University, Qld, Gold Coast, 4222 Australia; 3 King’s College Dental Institute, London, UK; 4 Griffith University, 170 Kessels Road, Nathan Campus, Qld, Brisbane, 4111 Australia; 5 Rural Clinical School, The University of Queensland, 152 West St, South Toowoomba, Qld, Toowoomba, 4350 Australia; 6 Faculty of Health, Queensland University of Technology, Victoria Park Road, Kelvin Grove, Qld, Brisbane, 4509 Australia

**Keywords:** Oral health, Aboriginal, Indigenous

## Abstract

**Background:**

Many factors influence how a person experiences oral health and how such experiences may facilitate supportive oral health behaviours. Women in particular face different challenges due to their environment, responsibilities and physiological differences to men. Within Australia, Aboriginal and Torres Strait Islander women are reported to have poorer oral health and are faced with additional barriers to supporting their oral health compared with non-Indigenous women. The objective of this paper is to report the experiences and perceptions of oral health from the perspective of urban, Aboriginal and Torres Strait Islander women.

**Methods:**

The present data derive from a descriptive study that used yarning circles and face-to-face interviews with women who were mothers/carers of urban, Aboriginal and/or Torres Strait Islander children. This was a qualitative study to investigate the impact of child oral health on families. Participants used the opportunity to share their own personal experiences of oral health as women, thus providing data for the present analyses. Information collected was transcribed and analysed thematically.

**Results:**

Twenty women shared their personal narratives on the topic of oral health which were reflective of different time points in their life: growing up, as an adult and as a mother/carer. Although women are trying to support their oral health across their life-course, they face a number of barriers, including a lack of information and the costs of accessing dental care. The teenage years and pregnancy were reported as important time periods for oral health support.

**Conclusions:**

To improve the oral health of Indigenous Australian women, policymakers must consider the barriers reported by women and critically review current oral health information and services. Current oral health services are financially out of reach for Indigenous Australian women and there is not sufficient or appropriate, oral information across the life-course.

## Background

Basic requirements for good oral health are, in general, similar across the life span: keep the mouth clean, use fluoride, limit sugar, avoid drugs and alcohol, and visit the dentist regularly [1]. Yet, despite the relatively homogenous recommendations for good oral health, many experience oral health differently. Our environment, life experiences, socio-economic status, overall health, age and gender can shape how we experience oral health and practice oral health behaviours [2, 3].

Whilst Aboriginal and Torres Strait Islander (hereafter respectfully referred to as Indigenous) Australians are reported to experience worse oral health than non-Indigenous Australians [4], little has been published in understanding oral health from the personal perspective of Indigenous Australians [4]. Likewise, little is known about how Indigenous women and mothers experience and perceive oral health, for themselves and for their family [5]. ‘Mothers’ specifically represent a sub-population that can have oral health experiences unique to their role [6]. Oral health can be influenced by behaviours related to women’s traditional gender roles such as being the primary carer of children and responsible for domestic duties which include but are not limited to grocery shopping, cooking meals and facilitating family health care [7, 8]. The intensity of these roles can be heightened if women are single parents. Within Australia, single mothers represent 82% of single parent families and experience more financial stress than their male counterparts [9]. In general, women are more likely to avoid the dentist because of the cost than men [10]. They are also physiologically different to men and can experience numerous hormonal changes during their lifespan which interact with their oral health status. The fluctuation of sex hormones (estrogen and progesterone) change the oral environment and can increase inflammation and bacterial colonisation, putting women at increased risk of caries and periodontitis amongst other conditions [11].

Understanding how women experience oral health across their life course has important implications, not only for them, but for those they care for as well. Poor oral health during pregnancy has been linked to low birth weight, preeclampsia and preterm birth [12] and maternal social determinants influence health-literacy, health-seeking behaviours, dietary knowledge and family oral health habits [13–16].

Studies suggest there are a number of barriers to oral health experienced by Indigenous Australians [17]. These include avoiding preventative care because of the cost and difficulty of attending oral health services because of family responsibilities, distance and waiting times [17, 18]. Additional challenges include finding services that are family-friendly and culturally safe [5], and there is a mistrust of the health system and a fear of judgement by health professionals [18].

Understanding the personal experiences and perceptions of oral health from urban, Indigenous women is necessary in order to provide services that are accessible, engaging, culturally safe and lead to better health outcomes for themselves and their children. Thus, our primary objective is to honour the voices and describe the experiences of urban Indigenous women while yarning on the topic of oral health.

## Methods

### Methodology

This study was conducted in accordance with the World Medical Association Declaration of Helsinki 2013 [19] and guided by the principles of Participatory Action Research (PAR). PAR is in contrast to the historical positivism and paternalism of social research and instead endeavours to distribute power and engage participants as researchers themselves [20]. The central tenets of PAR come from a strong background of collaboration and advocacy, whereby research can be used to progress change and support social and community agendas [21].

### Setting and context

This study came about because of a community interest in oral health. The research group had initially proposed investigating respiratory health in collaboration with an Aboriginal owned and operated primary health care clinic in Caboolture, a northern suburb of Brisbane, Australia. Senior members of the community indicated that oral health ought to be researched as well because there were limited data available on the oral health of urban Indigenous families, and the community was unable to effectively advocate for additional oral health resources. In conjunction with the respiratory study, a quantitative oral health study investigating the prevalence of caries and the associated risk factors was undertaken, and the results published [22–25]. Additionally, a qualitative study was proposed to provide a voice to the participants and assist in understanding the impact child oral health was having on families.

The primary health clinic where the study took place had an Indigenous client population of approximately 60% and hosted general practitioners, Indigenous health workers and midwives. It was affiliated with a dental clinic within the same building complex. Dental services are not provided for under the Australian public health care funding system, Medicare [26], and as such attract an out-of-pocket fee for service. Although a public dental care system exists, it is stipulated by eligibility criteria which varies by Australian state and Territory [26]. In the state of Queensland, adults must have a concession card, which is provided on application by a national social services agency such as Centrelink or the Department of Veterans’ Affairs [26]. A range of preventative and treatment-based services are available for those eligible. However, the services are associated with considerable waiting times [26].

### Design

In August 2017, the qualitative study commenced with the aim of investigating the impact of child oral health on urban Indigenous families. The study utilised PAR principles and in collaboration with the researcher, two participants took part in coding and theming the data to answer the research question. The results were published [25]. However, in addition to the data relevant to the research question, participants also contributed data which was deemed unrelated (as determined by the participants and researcher) to the initial research question, but valuable to the participants and their narrative. According to the Australian Institute of Aboriginal and Torres Strait Islander Studies [27], one of the principles of ethical research with Indigenous Australian populations is that the “research outcomes should include specific results that respond to the needs and interests of Indigenous people” [27]. The participants, while providing data related to the initial research question, also provided qualitative data on topics that were of importance to them and their own oral health as women. This paper focuses on reporting those data.

### Participants

Women of all ages were eligible for the study if they were or had been the parent or carer of an Indigenous child under the age of five. Purposive sampling was used and participants were recruited from the previous respiratory and oral health studies [22–25] and through word-of-mouth at the Aboriginal-owned and operated primary health care clinic. Two researchers undertook recruitment. KH, PhD, is an Aboriginal woman and nurse, who had been the lead investigator in the respiratory study and worked at the clinic as a nurse and Aboriginal Health Worker. KB, BPubHlth, is a non-Indigenous Canadian/Australian woman, who had been working within the study community on a separate research project and was responsible for analysing the quantitative oral health data and facilitating the qualitative oral health study. Participants were given the option to have both researchers present if they preferred, or if comfortable, could arrange a time with the primary researcher (KB). The study protocol was explained verbally and through a written plain language statement. Signed consent was obtained. Participants were offered the choice to meet at the health clinic or at a location comfortable for both them and the researchers. Data collection ceased once no new information emerged in participants’ responses to the initial research question: the impact of child oral health on families.

### Data collection

Yarning is an accepted method of sharing information and communicating within Aboriginal culture [28]. Yarning is not unlike a conversation as determined by Western definitions, whereby a story may be used to share information or knowledge. However, it arguably carries more significance within Aboriginal culture, and is considered a cultural practice. This is because, as has been described by Bessarab and Ng’andu [28] oral traditions have historically been the main form of transmitting knowledge and information within many Indigenous cultures, including Australia. Within a research context, yarning aims to mitigate the power imbalance between researcher and participant, which can come with an interview or conversation that has a research motive. Yarning places value on the participants knowledge and expertise and is an open process of sharing between the participant and researcher. This process is supported by the relationship developed between the researcher and participants before the research yarn takes place [28]. Bessarab and Ng’andu explain this as social yarning and involves getting to know a person through discussions unrelated to the research [28]. In this study, the researcher, KH, had already established relationships with the majority of participants from working in the clinic and facilitating the other studies. KH was able to introduce researcher, KB, to many of the study participants and begin establishing a relationship outside of the study. This meant that by the time the researchers and participants had scheduled the research yarn, there was a familiarity between primary researcher and participants. When the yarns took place, to establish rapport and provide additional context for the participants, researcher, KB, shared her own experiences of oral health, both as a young girl and as the mother to two boys. The yarns began with a starting question such as, “can you tell me about what oral health is like for your family?”. In this process, participants shared their thoughts related to the initial research question concerning the impact of child oral health on families, as well as their own personal experiences of oral health as individuals; sharing what oral health was like for them as children and throughout their life as they became mothers. All sessions were recorded with a digital audio recorder (Philips Digital Voice Tracer LFH662, Korea).

### Analyses

The information gathered was transcribed by two authors (KB and KH) and entered into the software package NVivo 11.4 (QSR International Pty Ltd. Version 11.4.1.1064). KB was responsible for facilitating the analysis and KH and MT were available for consultation and guidance. Analysis was guided by Braun and Clarke’s ‘6 Steps’ [29], which does not use a specific lens or theory to guide the analytical process but provides a framework to follow [29]. This includes becoming familiar with the transcripts, initial coding, searching for themes, reviewing themes, defining the themes and writing them up [29]. This process was supplemented by participants’ contribution to the coding and theming steps. Participant involvement in qualitative analysis is necessary to uphold collaborative research agreements, as well as a method to support the validity of the data [21, 30]. Upon reviewing the transcripts, the researcher, KB, inductively coded the data and printed the coded transcripts to paper. These codes were informed by personal notes made after the yarning sessions and informal discussions with KH. The transcripts were then brought to two participants on separate occasions who had expressed interest in participating in the analysis process. All other participants were offered the opportunity and either declined or were unreachable upon three contact attempts. The researcher and participants used the transcripts, along with blank pieces of paper (‘post-it notes’) for any additional information and to place the codes into initial themes. This process was iterative and prompted by a discussion of the yarning sessions and the participant and researcher sharing their ideas about the data that arose and potential themes. For instance, some concepts were not repeated individually within the group sessions, but consensus was given by participants by way of agreement and affirmations. Like codes were grouped together and once established, it was discussed whether the themes were relevant or not in terms of the research question. This was both a tactile and verbal collaboration that aimed to acknowledge the participants as experts on their own lives and counteract the sub-conscious bias that can occur with non-Indigenous participation in research [31]. Some themes were leftover in the analysis process and although they did not fit the original research question: ‘what is the impact of child oral health on urban Indigenous families?’ (results of which have been published separately) [25], the participants and researchers, KH and KB, agreed that they were representative of the participants’ personal stories as women in addition to their experiences with child oral health and should be considered for publication in their own right. These themes were discussed and finalised by KH and KB for publication.

## Results

Twelve individual interviews and 2 yarning groups with 3 and 5 people respectively, were conducted. All participants were mothers with the exception of one aunty. Of the people approached, no one declined participation. The majority of the participants were recruited from the larger study population attending the health clinic. According to previously collected data, the families in the study population had an average annual income of <$37,000 (US) and 85.5% of mothers were unemployed [24]. Both researchers were present for the group sessions and for 3 of the interviews. The other 9 interviews were conducted by the non-Indigenous researcher independently. Yarns took place at a number of different locations including: the clinic, church and school halls, cafes and local parks. The duration of the yarns ranged between 35 min and 2 h. Along with discussions regarding the impact of child oral health, which have been previously published [25], participants shared their personal narratives on the topic of oral health. The additional discourse is reflective of the yarning process and the participant focused approach of the study. Congruence was observed in participant responses, with women sharing similar experiences and perceptions in both the interviews and group yarning sessions. The themes raised were associated with three stages of life: growing up, as an adult and as a mother and are organised under each stage as shown in Table 1.

**Table 1:** Stages of life and themes raised during yarning

Growing up	As an adult	As a mother
• Teenage perceptions • Fluoride • Traditional approaches • Diet • Negative impacts	• Disease • Traumatic past experience • Cost • Tooth loss and wellbeing	• Pregnancy • Diet • Lack of information • Recommendations for health promotion

### Oral health growing up

Childhood experiences of oral health varied for participants. The geographic location of participants when they were growing up, along with their age and resources of their parents all influenced their experiences. Most participants indicated that they have more information now than their parents did; cleaning teeth was an accepted practice, but the regularity and approach varied amongst families. Some families were quite ‘strict’, and parents supervised tooth brushing at least once a day. Others indicated that their parents provided the supplies (tooth-brush and toothpaste), but didn’t necessarily monitor their oral hygiene habits. Some participants grew up in regional and remote areas and did not have running water or electric power which acted as a structural barrier to regular oral hygiene habits, even if they did have the personal resources.

*We weren’t taught about mouthwash or flossing, we were just taught about brushing, we didn’t know it was about your gums too, I never remember that. But now we learn all that, you know, brush your gums, and clean your whole mouth. Yeah, when I was a teenager, I didn’t give a toss, but then you come to full circle into an adult and you start taking care of it, you remember ‘oh yeah that’s why’, and you start to do things. (Interview – Mum A)*

#### Teenage perceptions

Of those who insisted their parents were ‘strict’ about oral health and regimented tooth brushing, there was not a consensus on whether this influenced their own approach to oral health care in their youth. Many participants remarked that oral health did not become a priority for them until they themselves were adults and started having problems with their oral health. Many participants acknowledged that they did not practice regular oral hygiene habits as teenagers and remember actively disliking having to maintain their oral health. However, most did participate in school dental service visits, which in Queensland are designed to screen all children and render all dentally fit: this was their primary source of professional dental education.

*I hated it. There was a period of time where I never used a tooth-brush at all, I just used a towel or whatever I had and went like this (action of rubbing towel across teeth with fingers), but now I brush all the time because I don’t want false teeth. (Group 1 – Mum 1)*

#### Fluoride

Many participants remembered attending school dental services regularly and the use of fluoride tablets administered by the school or their parents. The use of fluoride was generally accepted as a good thing and participants recalled fluoride either being in the water naturally, being added or receiving tablets. Fluoride tablets, along with disclosing tablets (chewable tablets with a vegetable-based dye that colours dental plaque) seemed to evoke a lot of detailed memories for participants and they shared stories of learning about plaque and the tablets they were given to demonstrate how to brush effectively. These tablets caused some embarrassment but were indelible in participant’s memories as a kind of ‘rite of passage’ during childhood.

*I remember one time because my front tooth crossed over the other, it would be really dark and I hated it and we would have to take them home and we would have a pink one at home and you would have to chew on it before you brushed your teeth and it would show (the plaque) then you would brush your teeth to see if you were getting it. (Group 1 – Mum 2)*

#### Traditional approaches

Traditional approaches to oral health such as the use of charcoal for cleaning teeth was discussed by a number of older participants; younger participants also indicated that they remembered their parents sharing traditional teeth cleaning methods with them and had an interest in reviving traditional methods for themselves. One participant had acknowledged how oral health had come ‘full circle’ with the availability of charcoal toothpastes in conventional stores. Participants remembered fondly that in addition to teeth cleaning, their parents and elders had used charcoal in a number of household products. This was attributed to cultural practices, but participants also acknowledged that cost was a factor in utilising alternative methods; often conventional items were more costly than home remedies.

*They cooked in the charcoal, and they didn’t shake it off, they just ate it. I can remember going fishing they chucked the fish on the coals. I can remember sitting there spitting out charcoal, you know what I mean about the crunchy gritty bits, but that’s how they lived off the land. (Group 2 – Mum 3)*

#### Diet

Along with more culturally specific practices such as the use of charcoal, participants reminisced about the difference in their diets when they were young. Acknowledging that foods were less processed in their childhood and they had less exposure to sugars.

*Fluoride was in the water, so we had the healthiest teeth in the cemetery. But being a child back in my day, my folks and I didn’t have a lot of money, so we ate a lot of fruit and mum killed her own cows and stuff, and we did all that. We never went to the shops to buy anything, because mum milked the cows, the goats. We used to eat chook eggs, the duck eggs, you know. (Interview – Mum B)*

#### Negatives impacts to oral health

Not all past practices were as well regarded as the use of charcoal and home-cooking. Participants shared that they felt that the past social acceptance of smoking likely contributed negatively to their oral health as an adult. As did a lack of preventative dentistry and some dietary habits, such as having baby bottles containing tea or a carbonated, high sugar, beverage. Participants were cognisant of the yellowing of their teeth from a young age and this was seen as undesirable, even when they were young.

*You know one thing I feel really strongly about now that I know, is we were brought up with mum and dad smoking in the house. Mum and dad smoking in the car. Mum and dad smoking around children. You know what I mean, and then I look at my teeth and think to myself how yellow they are. And when I go to the dentist, they ask how long have you been smoking: I have never smoked in my life. You know what I mean and then I look back at it and think to myself, like for them to say smoking causes yellowness, and you have that yellow look on your teeth. (Group 2 – Mum 3)*

### Oral health as an adult

Despite the varied experiences of participants as children, whether they had practiced regular preventative oral health habits or had less structured habits, most indicated that as adults they had experienced poor oral health. Some participants acknowledged that their experience was likely a result of their poor oral health as teenagers. Whereas others felt that they had poor oral health despite the efforts they made to support good oral health. Disease, traumatic past dental experiences and cost were raised as impediments to their current oral health, with tooth loss and its effect on well-being also a commonly shared concern.

#### Disease

A number of participants explained that good oral health was taken away from them by another health condition. Conditions discussed included diabetes, cancer and hypothyroidism. There was a sense in the discussion that they had no agency over their oral health because of their systemic disease. A shared sentiment was that their poor oral health was ‘coming from the inside’ and that externally it didn’t matter what they tried, their oral health seemed to be out of their hands.

*For me I had the perfect teeth, you know, and it wasn’t until I got diabetes and I’m fanatical with brushing teeth and mouthwash and flossing, you know, but because of diabetes and stuff like that - I mean don’t believe everything you hear about: ‘if you don’t do this and you don’t do that, this is what’s going to happen’. You can do all the right things and still end up with no teeth. Like I’ve got no teeth because I got really bad infections from my wisdom teeth. I’ve got no molars and I had root canal here, so you can be fanatical as you want. (Group 2 – Mum 2)*

#### Traumatic past dental experiences

A number of participants shared ‘dental horror stories’, where their oral health and well-being was negatively impacted during dental visits and this made them reluctant to ever attend the dentist again. Some participants readily articulated that they had a dental phobia, whereas others reflected that the fear of pain and their childhood memories of the dentist put them off attending.

*And then I got wisdom teeth down and the dentist lady used my good tooth to anchor off and she shattered it. So I still have bits of jaw bone come through, yeah so I’m picking bone out every now and then. I’m picking bone out going, where did this come from? (Interview – Mum B)*

#### Cost

There was a consensus amongst participants that the cost of dental care for adults was prohibitive. Many shared anecdotes of the dental restorative work they required but had put off indefinitely because they could not afford it. Participants agreed that they would just wait until there was an issue of pain or loss of function and going to the dentist was no longer optional. For some participants, there was the notion that the dentist was not a welcome place unless you had the work done that was recommended, so why bother going for prevention. One participant shared how she was saving up to go overseas for dental care, hoping to have dentures made as the cost of keeping her teeth in Australia was too much.

*I’ve got extras health insurance and I still don’t to go unless I have to and there is something going on. I’m supposed to have a plate to wear when I am sleeping but that thing costs like $400-$500 dollars. (Group 2 – Mum 1)*

#### Tooth loss

Tooth loss was a commonly shared concern amongst participants. As was the impact on well-being and confidence when teeth were removed or damaged. Participants explained that tooth loss influenced the way they ate, drank, spoke, smiled and laughed. One participant shared how it had affected her ability to find work because of judgement from potential employers.

*Your face changes, even the way you talk, even just rinsing your mouth after you brush your teeth, you know water spurts out. You have to be careful when you are out drinking or eating. I work in a high school and I don’t even laugh anymore because of it. (Group 1 – Mum 3)*

Few reasons for tooth loss were discussed specifically. As mentioned previously, some participants shared that their overall health contributed to their poor oral health and others also shared how pregnancy contributed to tooth loss. However, some participants indicated that tooth loss was a result of their limited choices in dental care. Suggesting that given more options for oral health services, they could have kept their teeth.

*Going to the dentist nowadays it is cheaper to get a tooth taken out than filled most of the time. (Group 1 – Mum 2)*

### Oral health as a mother

All participants indicated that their oral health changed when they became a mother. Most insisted there were noticeable physiological changes. Others suggested that the change came from the deficit in time and energy associated with becoming a mother: their focus was moved to their children. As has been reported previously [25], supporting children’s oral health can be stressful and mothers have multiple priorities to juggle including the oral health of their child.

#### Pregnancy

Participants had a lot to share on the topic of pregnancy and oral health. It seemed accepted and commonplace that a woman’s oral health would be impacted with pregnancy. A couple of participants shared some interpretation of the proverb: ‘gain a child, lose a tooth’. There wasn’t a lot of negativity in the yarning, it was just accepted as a fact of motherhood that with children, came poor oral health and even tooth loss. Participants described being drained of nutrients and their bodies giving everything to the baby. No comments were made regarding maternal oral health education or information and so it is difficult to comment whether participants felt that they should have had been provided with more information during their prenatal period.

*I think all of it too is having kids, they are just drawing everything out. They just suck the life out of you. They do, because it is all nutrients, the baby is taking everything, you know everything in your gums. That’s when I first started having trouble with my teeth. (Interview – Mum C)*

*I actually had pretty good teeth up until 19 years ago. And yeah it was true, the minute I fell pregnant I started having issues with my teeth. I was 18 and yeah that’s when I first started having problems. I mean I had fillings all though school, but that’s when they really started deteriorating and the first pulled. (Interview – Mum D)*

#### Diet

All participants were aware of the damage that sugar could cause to teeth and shared their approaches to finding balance with their child’s sugar consumption. Many participants shared how it had been an ongoing education for them learning about the effects of sugar on teeth. One participant acknowledged that it had become easier since the obesity epidemic to understand the negative impact of sugar, but still felt there were a lot of mixed messages in society.

*The biggest thing for me, I mean my kids still have sugar, they still have soft drink, they still have lollies, chips, ice-blocks. I think it’s all about kind of balancing. You know we might have a treat day, and then the next day we will cut back a little bit, but I’m not so concerned about that. Or that’s not my main concern. But I’m not going to put her to bed with a coke, you know, she is still being breastfed, she’s not having a lot of that food, so I’m not too worried. (Interview - Mum E)*

*It comes down to your dietary intake, eating your fruit and vegies, if they don’t like what they see in solid, I will blend it and put it on pizza bases or in spaghetti bolognaise. They are going to get it one way or another. (Interview – Mum F)*

#### Lack of information

A number of participants explained that they either ‘learned the hard way’ or had to go out and get information for themselves because of a lack of oral health information, specifically regarding child oral health. One participant in a group yarn shared that she felt that many people in her community did not have enough information and she had never seen any culturally specific information, the group agreed there was nothing available. This sentiment was also shared in other individual yarns with participants commenting on how they perceived the lack of information for their community.

*You still see it now when you’re out walking, kids with parents, the kids are just handed a bottle of coke. (Interview – Mum A)*

A few participants shared that they had ‘done the wrong thing’ and gave their children milk or juice in bottles or sippy cups as they got older, believing that milk and juice were healthy.

*I had to go research it myself, there’s not a lot of parents that get to have the internet and stuff. Like I can’t get on the internet. It’s not like old-school where you can go to an encyclopaedia. Yeah maybe through the school, there is not enough in the school*. *(Interview – Mum B)*

#### Recommendations for health promotion

There were mixed feelings about oral health promotion. Most participants agreed that they had more information than their parents, but they also felt that that in some instances some of the approaches that were used when they were children were more beneficial and got a stronger message across than what was available now. For instance, the use of the tablets to colour plaque, school-based interventions and commercials on television. A number of participants lamented that Australian children no longer had a ‘Mrs Marsh’ to learn from. During the ‘70s and ‘80s, television commercials featuring ‘Mrs Marsh’ were aired in Australia, promoting oral health behaviours. A shared sentiment was that more information was needed in schools and parents needed more clear information when children were very young. A few participants shared that it would be good if oral health was addressed when child health nurses came for infant checks or when they had health checks done. Further integration of both primary health care and dental services was raised specifically by one interview participant.

*I think it (dental) could be more into the medical side of things, like medical centres as well because you know it seems so separate. You know you got the dental side and the medical side, and I think they really need to push it, you know when they do the health checks, they really don’t check too much about the dental. (Interview – Mum E)*

*I think to educate kids, especially little, because that is where it starts. You need to start these little jingles up again about brushing your teeth. We do it with hand washing, do it with teeth, you know. Take this jingle back home to mum, like “slip slop slap”. And then maybe with the preppies to year 3, create a person that will come in and dress up as a tooth and show them how to brush their teeth, you know. (Group 2 – Mum 3)*

*I also think there does need to be more education for the parent, particularly in those early years. If they don’t have good dental hygiene, they are not going to be able to teach the kids well and for them just waiting until school age, there needs to be something out there for the parents. (Group 2 – Mum 1)*

In addition to information, some participants acknowledged that limited oral health resources were a problem for families.

*Your mouth is, you know talking, eating, and drinking. There is just so much involved around it. I used to do Indigenous support in high schools and we used to have a program focusing on general well-being and puberty and body image, and it was focused on your hygiene and we would sit down and talk to the kids as something as simple as: ‘it was important to brush your teeth twice day’. I mean some of these kids didn’t even have a toothbrush. Well this is when I was working in Toowoomba and we would have to fund it ourselves. We would have to outsource toothbrushes and toothpastes and you know little deodorants and soaps and you would be surprised how many Indigenous kids did not have the equipment, the means to get that and it was really sad. (Interview – Mum E)*

## Discussion

Our study contributes to a growing body of research reporting the experiences and perceptions of Indigenous Australian women on the topic of oral health. Our previous publication [25] from this dataset reported the impact of child oral health on families from the perspective of mothers. The focus of this paper was to present the voice of the mother speaking for herself as a woman and individual, not limited to the motherhood aspect of her identity or spokesperson for her family. Adding a generational context to our previous publication, this paper sheds light on how Indigenous Australian women experience oral health during different stages of their life: growing up, as an adult and as a mother.

The present findings from Queensland align with other research [5, 18, 32] which has qualitatively investigated oral health experiences of Indigenous Australian women in Western Australia and South Australia. The research suggests that current oral health policies are insufficient in supporting Indigenous Australian women across their life course. Indigenous Australian women care about their oral health and they would like more consistent and culturally appropriate information to support their oral health behaviours from birth into adulthood, as well as more options to access and pay for professional oral health care should they need it as adults [5, 18, 32] .

Oral health policy and health promotion in Australia has historically been inconsistent. Unlike the public medical care funding system, Medicare, there has been no sustained, publicly funded, oral health program that provides subsidised dental care for all ages. Past and present government subsidised dental services have largely been targeted at school-aged children and, as our participants have shared, the effect of these programs is not necessarily long-lasting. There needs to be more consistent opportunities for information throughout the lifespan, so information can be passed down generationally and supportive oral health habits perpetuated.

Similarly, consideration must be given to the wider social contexts in which Indigenous Australians are trying to maintain their oral health. The effects of colonization have contributed to widespread disadvantage and present policies do not consider these ‘structural barriers’ to oral health [17, 33]. The cost of oral health is not limited to services, but to the environment in which oral health behaviours occur, including factors such as the cost of a healthy diet, supporting overall health and accessing education which supports health literacy [17, 18, 33]. Using hindsight, participants in this study have highlighted some of the challenges in supporting oral health within their own environments; providing insight into the gaps in service provision and missed opportunities for health information and promotion.

According to our findings, previous policies have fallen short in supporting older teenagers. Participants indicated feelings of apathy during their teenage years toward their oral health, which did not resolve until they reached adulthood and began having problems with their teeth. The School Dental Service, the first federally funded program for school children founded in 1973 provided for children aged 5–15 years. Providing Australian children regular opportunities for professional screening and care, the program was devolved to State and Territory government responsibility in the 1980s and is presently only active in select schools [34]. The percentage of children attending school dental services dropped considerably (54 to 21%) during the period of 1994 to 2013 [26]. Likewise, subsequent federal programs such as the Teen Dental Program (2007) [34] and the current Child Dental Benefits Scheme (CDBS), are not available in all schools, but can be utilised during public and/or private dental consultations. The CDBS provides all eligible Australian children aged 2–17 with $1000 every 2 years towards their oral health [26]. Whilst it provides financial support for families, it is dependent on parent/carer engagement with a dental practice if one is not available at their child’s school. We are unable to comment on how our participants have received the new policy as it was only newly introduced when the study was conducted. Yet, it was clear that more information and interventions from schools would be welcome. An evaluation of the uptake of CDBS by all children and teenagers is required to determine whether Indigenous Australians are accessing the service given the inconsistency in service provision and adult attendance to dental services.

Participants in this study had strong memories of using disclosing tablets to demonstrate plaque as children and Joanne Kerr advocates that this approach could be used with teenagers again [35]. Kerr points out that while disclosing tablets may be unsophisticated and typically used with primary school children [35], our study suggests they are readily recalled and considered useful. Research suggests that aesthetics is a key motivator for teens in terms of oral health [36, 37], with teens caring more about the appearance and smell of their mouth than the actual health status [37, 38]. We were unable to find any studies that had investigated the efficacy of disclosing tablets with teenagers specifically, and studies examining their effectiveness in this population are warranted.

Women in our study reported that their oral health declined not only with their age, but with their transition to motherhood; suggesting that their pregnancies had physiological effects on their oral health. Pregnancy can affect oral health [12]. However, the notion that pregnancy is threatening to oral health, while not unfounded [12], is perhaps misleading. The misconception that poor oral health is inevitable when you have children is not uncommon, [39] but it is indicative that oral health information is not reaching Indigenous Australian women. Antenatal care represents an opportunity to commence a generational cycle of oral health education. There was a consensus, that women are not attending the dentist themselves regularly and feel like there is little information coming to them from other sources. Our findings support other studies [6, 40–42] which suggest that oral health care needs to be embedded into antenatal care. However, there remain considerable barriers within the current Australian policy landscape for this to happen. Although pilot projects [6, 40, 43] have demonstrated that midwives are cognisant of the importance of oral health for their patients, they feel they do not have the time or skills to provide adequate care. At present, to our knowledge there is only one midwifery course in Australia offering formal oral health education to students [44]. While it shows promise in increasing the oral health promotion skills of midwives, it is limited in its ability to support the ‘next step’ in the care process [44]. Midwives indicated that with education they feel confident in making referrals to dentists, but it means little when women are not able to access those services [44]. Like our participants, midwives report that cost is a major barrier to accessing dental services. Free dental clinics for pregnant women and an online referral system have been proposed by midwives to overcome these barriers [44] and our findings indicate that these services would be well-received.

Unfortunately, subsidised oral health services remain limited for adults in Australia, including pregnant women. Within Queensland, public oral health services are only available for concession card holders and are associated with significant waiting periods [26]. While we did not determine whether all of our participants were aware of the services available to them, it was clear that they felt they had limited options and agency over their oral health. At present, as an adult in Australia if you are unable to afford a private dentist, there are few opportunities to receive oral health information or advice. Many Indigenous Australians participate in general health checks, dubbed ‘715 s’ as per the Medicare Benefits Schedule billing code [45]. These health checks are specifically for Indigenous Australians and meant to evaluate and support all aspects of health, including oral health. According to the ‘715’ criteria, patients are to receive an oral examination and a referral to a dental professional if further investigations or treatment is needed. Our study and others [46, 47] suggest that the oral health criteria are not routinely being met. Bailie et al. [46] posits that services, such as oral health examinations, that do not have clear referral pathways are less likely to take place during health checks [46].

In the absence of referral options, discussion and education regarding oral health should be prioritised. The common-risk factor approach has been proposed as a way of improving the oral health of Indigenous Australians [48]. Given many health conditions share a common set of risk-factors to oral health, education could be bundled, and advice given holistically [48]. Yet this approach is unlikely to be achieved while treatment is prioritised before prevention. Our study lends weight to the growing call for radical reforms to the current model of oral health [49, 50]. Watt et al. and Cohen et al. have indicated that the present treatment focus on individuals is harming those that are most vulnerable and unable to afford care [49, 50]. Indeed, all our participants reported poor oral health as adults and within the wider study population, 75.5% of participants had self-reported decayed, missing or filled teeth [24]. In other studies, Indigenous adults have reported considerable negative oral health issues including high levels of caries, tooth loss and pain [4]; this is supported by national data that demonstrates a disparity in oral health between Indigenous and non-Indigenous people [26]. Watt et al. and Cohen et al. argue that within dentistry, prevention should be prioritised, and the responsibility of oral health disseminated amongst a primary care team [49, 50]. In addition to increasing the accessibility of oral health services, this approach would likely improve the regularity at which Indigenous Australian women received oral health information and education. As shared previously, oral health information is not getting to Indigenous Australian women and they feel that society would benefit from a greater diversity of oral health promotion, perhaps even reviving Mrs. Marsh. Recent analysis of oral health messages in Australia mass media shares their sentiment and researchers suggest that there is a ‘contemporary crisis in population level oral health promotion’ [51], with oral health promotion severely lacking social context and usable content. These insights are valuable for finding a way forward in terms of health promotion and suggest there is an audience for culturally appropriate and potentially co-designed health promotion materials.

## Limitations

As the findings described in this paper are opportunistic, it is possible that they do not adequately reflect the breadth of data that could have been reported had there been a targeted research question that queried women’s experiences across the life course. Further, determining if women’s perceptions changed with age would have been valuable. However, the extent of the data collected and the commonality in the data between participants of different ages suggest that the themes raised are topics of importance to the participants and as such should be considered nonetheless. Another potential limitation to the findings is the participation of a non-Indigenous Australian as the primary researcher. Although there appeared to be no difference in the topics raised between yarnings, this is only the perception of the researcher and participation of a non-Indigenous Australian may have influenced the comfortability of the participants.

## Conclusions

There is limited qualitative data [5, 18, 32] reporting the oral health experiences and perspectives of Indigenous Australian women. This study highlights that Indigenous Australian women are experiencing similar challenges to their counterparts in Western Australian and South Australia. The research demonstrates that both past and present oral health policies have been insufficient in adequately supporting the oral health of Indigenous Australian women. Current policies are short-sighted and do not consider the economic barriers to good oral health within a treatment-focused oral health system. Nor do they provide enough opportunities for oral health education across the life course. Indigenous Australian women have their own views of what they would like to see in terms of information and services, and policy-makers are urged to consider the available evidence to develop and facilitate future oral health care programs.

## Data Availability

Study data and materials may be made available on request with the appropriate human research ethics committee approval and with the consent of the participating community as required by Australian criteria for research within Indigenous communities.
